# A telemedicine meditation intervention for people with multiple sclerosis and their caregivers: study protocol for a randomized controlled trial

**DOI:** 10.1186/s13063-015-1136-9

**Published:** 2016-01-04

**Authors:** Cesare Cavalera, Francesco Pagnini, Marco Rovaris, Laura Mendozzi, Luigi Pugnetti, Massimo Garegnani, Enrico Molinari

**Affiliations:** Department of Psychology, Catholic University of the Sacred Heart of Milan, via Nirone 15 – 20123, Milan, Italy; Multiple Sclerosis Rehabilitation Unit, Istituto di Ricovero e Cura a Carattere Scientifico S. Maria Nascente, don C. Gnocchi Foundation, Via Capecelatro 66, Milan, 20148 Italy; Laboratory of Clinical Neurophysiology, Istituto di Ricovero e Cura a Carattere Scientifico S. Maria Nascente, don C. Gnocchi Foundation, Via Capecelatro 66, Milan, 20148 Italy

**Keywords:** meditation, mindfulness, multiple sclerosis, telemedicine

## Abstract

**Background:**

Mindfulness-based interventions, modified and shortened versions of meditation teachings, have proved to be effective in the improvement of quality of life in many clinical conditions, including chronic diseases. Preliminary results available in the literature and in clinical experience indicate a high potential for this treatment for the reduction of psychological suffering in people with chronic diseases.

**Methods/Design:**

This randomized controlled trial will investigate the impact of a multiple sclerosis (MS) specific telemedicine meditation intervention on the quality of life of people with multiple sclerosis and their caregivers. This trial will recruit 120 patients, men and women, with a diagnosis of relapsing-remitting or secondary progressive MS and their caregivers to participate in a 2-month intervention. Patients will undergo assessments of quality of life, anxiety, depression, quality of sleep, mindfulness and fatigue levels conducted at baseline, at week 8 (conclusion of the intervention) and at week 27 (6 months follow-up). Caregivers will complete assessments conducted at the same time for the same areas, plus caregiver burden. The intervention condition will consist of 2 hours/week of online meditation in a group setting led by a trainer, plus 1 hour/week of individual exercises. The control condition will incorporate a psycho-education online program and will require the same contact time commitment as the intervention condition.

**Discussion:**

Primary outcome measures will consist of assessments of quality of life, anxiety, and depression level. Assessments of mindfulness level, quality of sleep and fatigue level will be considered secondary outcome measures.

This investigation will increase understanding of the role of meditation as part of a treatment plan for people with MS and their caregivers. Overall, this study design has the potential to lead to effective meditation intervention strategies for this population and improve their quality of life.

**Trial registration:**

Clinical Trials Register NCT02364505. https://clinicaltrials.gov/ct2/show/NCT02364505

## Background

Multiple sclerosis (MS) is a chronic disease of the central nervous system that results in worse quality of life than that experience by persons living with other chronic diseases [1]. The unpredictable and variable nature of MS makes it particularly difficult to accept; moreover, the grim prognosis and the added unpredictability of day-to-day health in relapse-remitting MS and side effects of medication [2] greatly impact quality of life and quality of sleep. Fatigue, anxiety, and depressive features are often reported by people who have MS [3–5]. In particular, lifetime prevalence of major depressive disorder is approximately 50 % [6]. Moreover, the impact of care for this kind of disease on family caregivers can be very high: caregivers often suffer from sleeplessness, fatigue, anxiety, depression, and impaired immunological responses [7].

Psychological interventions may be effective in the reduction of distress, anxiety, and depression, also reducing MS symptoms [8], but further exploration is required. Meditation teachings have proved effective in the improvement of quality of life in many clinical conditions [9–11], including chronic diseases [12, 13], although accessing it is a challenge for a part of the population [14, 15]. In a previous study, the use of a standard meditation protocol was found to promote an improvement in psychological well-being of people with MS [16]. Patients with MS who were assigned to meditation condition experience an improved quality of life, and reduced levels of fatigue and depression up to 6 months after the intervention. Study limits included the fact that the protocol was designed for the general population, not for the clinical peculiarities experienced by people with MS. Moreover, the clinical needs and problems of these people might prevent full participation in meetings. Lastly, MS caregivers may benefit from this treatment, but neither this study, nor any other study, has yet explored this option in depth.

Available data suggest a relevant clinical potential for an online meditation treatment for people with MS and their caregivers. While treatments provided through technological platforms (telemedicine in particular) have been developed for other populations in recent years [17], few MS-related programs take advantage of the internet and related telecommunication platforms. Some promising efforts have begun: results from a recent pilot study [18] suggested that a problem-solving 5-week internet-based self-help program benefited MS patients with depressive symptoms.

### Aims

The main study aims are as follows:

1. To create and test a multimedia software that can teach meditation at home, following the specific requirements of people with MS.
2. To investigate the effects of this telemedicine mindfulness training on the improvement of quality of life of MS caregivers. While protocol changes will be made according to MS peculiarities, the resulting protocol will also be suitable for healthy populations, including MS caregivers.
3. To assess whether patients undergoing this meditation protocol show a change in their sleep patterns, which may reflect an improvement in their quality of life.

## Methods/design

### Study design

The study design of the randomized controlled trial is composed of two arms (experimental and control groups, both of 8 weeks duration) and three assessments (2 × 3 design): baseline, directly after treatment, and 6-months follow-up. Each participant will provide informed consent. Ethical committees from both the Catholic University of Milan (reference 19-12-2013) and the Fondazione Don Gnocchi (reference FDG_9.4.14) have approved the project. Results will be published in both scientific and general-audience media.

### Meditation and control protocols and software development

The first part of the project is dedicated to the adaptation of the meditation protocol to MS requirements and clinical peculiarities, the creation of the software, and a testing phase. To maximize its effectiveness [19], the meditation protocol will consider the potential impact of MS symptoms (e.g., numbness, fatigue, tingling), implementing meaningful changes to be more MS-related than the original mindfulness-based stress reduction protocol, for example, by implementing music meditation experiences and relaxation exercises. The protocol will be implemented using multimedia software, which will provide the learning and the practice of the intervention. It will be a course, provided with audio and video stimulations, with multiple interactions with the trainer. At the same time, an online control intervention will be implemented. This control intervention includes a psycho-education online program about MS, characterized by interviews, practical advice, and exercises [20]. The contact time commitment will be the same for both conditions.

### Software testing

After the development of the software, it will be tested for ergonomics and usability. Five patient–caregiver couples will receive the software and be instructed in its use. Their qualitative feedback, during the 2 months of the intervention and afterwards, will be useful in making changes that could improve the usability and reduce unexpected events (e.g., code program errors).

### Sample size and recruitment

We aim to recruit 120 people with MS together with their primary caregivers (therefore, the potential sample is up to 240 subjects; 120 patients and 120 caregivers).

Inclusion criteria for patients with MS will be:

- Diagnosis of relapsing-remitting or secondary progressive MS (divided into 60 relapsing-remitting and 60 secondary progressive during the recruitment procedure);
- Ability to communicate and to understand tasks;
- No change of disease-modifying treatment in the 3 months before the enrolment;
- No clinical relapses or use of steroid treatment during the 4 weeks before the enrolment;
- Availability of a personal computer, smartphone, or tablet (compatible with the software);
- Provided informed consent for study participation;
- Age >18 years.

Inclusion criteria for caregivers will be:

- Being a person who lives with the MS patient and provides him or her with the most care and assistance;
- Ability to communicate and to understand tasks;
- Availability of a personal computer, smartphone, or tablet (compatible with the software);
- Provided informed consent for study participation;
- Age >18 years.

Exclusion criteria (referred to both patients and caregivers) will be:

- Severe co-morbidity that would reduce life expectancy to less than one year (e.g., end-stage oncological diseases or severe cardiac dysfunction);
- Severe neuropsychological impairment (e.g., dementia), as indicated by testing below the fifth percentile in at least 3 of 6 dimensions of neuropsychological functioning tests (i.e., attention and concentration, processing speed, executive function, verbal memory, and verbal processing);
- Psychosis or dissociative disorders;
- Pregnancy.

Subjects will be recruited at the Multiple Sclerosis Center of the Fondazione Don Gnocchi Hospital, in Milan.

### Procedure

**Fig. 1:**
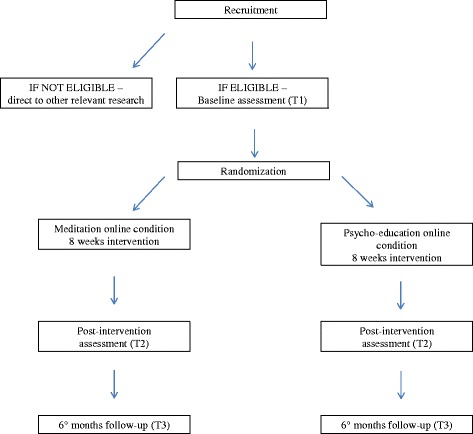
Overview of study procedure

Progression to the study is outlined in Fig. 1.

### Screening, assessments, and randomization

Screening will be conducted by doctors of the Multiple Sclerosis Center of the Fondazione Don Gnocchi Hospital, considering the inclusion and exclusion criteria.

Subsequently all subjects will be fully assessed at baseline (T1), after 2 months (T2, corresponding to the end of treatment) and at 6-months follow-up (T3).

Primary outcome measures will assess quality of life, together with depression and anxiety. Attained mindfulness level, quality of sleep, and participation level will be considered secondary outcome measures. Questionnaires will be self-reported, with online assessments (therefore accessible from subjects’ own computers). Subjects will be also assessed, with a qualitative approach (semi-structured interview), about possible side effects or adverse events of the treatment, at T2 and T3, by a psychologist. The interview will be conducted in person or via teleconference call (e.g., Skype).

Subsequently, randomization will be conducted by the principal investigator who will be fully blinded to all patient information, except the study identification numbers of patients. Patient–caregiver couples will be considered as single units for the randomization.

Subjects, consisting of patient–caregiver couples, will be randomly allocated into two groups (60 patients + their caregivers for each groups, with a stratification referred to subjects recruited for the instrumental assessment):

- Meditation telemedicine intervention, developed and tested.
- Control group, receiving psycho-education, utilizing a telemedicine approach.

The intervention phase will begin at the end of the pilot study (month 6) and will last 11 months.

In the mainframe of the general study, an objective evaluation of the impact of treatment on sleep disturbance and fatigue will be conducted with an instrumental assessment. During the software development and testing phases, we will consecutively recruit 40 subjects with MS (first recruitment round), with the same inclusion criteria, who report disturbed sleep. Each of these subjects will undergo actigraphy. The sensor will be worn by each subject for two consecutive weeks and will provide objective information about quality of sleep and daily activity, with synthetic data summarized by a computer software.

As soon as the intervention phase begins, these 40 subjects will be randomly allocated to one of the two groups and will undergo all the psychometric assessments, as for the other participants. These subjects will be assessed by actigraphy again, in a post-intervention assessment. The method will remain the same, with an identical timeframe (2 consecutive weeks).

### Measures

#### Self-report measures

The Multiple Sclerosis Quality of Life*-*54 questionnaire is a multidimensional health-related quality of life measure that combines both generic and MS-specific items in a single instrument [21]. It is composed of 12 subscales along with two summary scores, and two additional single-item measures. The subscales are: physical function, role limitations, physical, role limitations, emotional, pain, emotional well-being, energy, health perceptions, social function, cognitive function, health distress, overall quality of life, and sexual function. The scale has demonstrated good psychometric properties and has been translated into Italian [22]. This assessment will be completed only by patients.

The Medical Outcomes Study Short*-*Form*-*36 health survey will be used to assess caregivers’ quality of life. This is a 36-item scale constructed to survey health status and quality of life [23], that investigates eight health aspects: limitations in physical activities because of health problems; limitations in social activities because of physical or emotional problems; limitations in usual role activities because of physical health problems; bodily pain; general mental health; limitations in usual role activities because of emotional problems; vitality; general health perceptions. It is one of the most widely used instruments for the evaluation of quality of life in many countries in the world, including Italy [24].

The Hospital Anxiety and Depression Scale will be used to assess depression and anxiety levels of all the subjects. This comprises 14 items rated on a four-point Likert scale [25]. It is designed to screen for the presence and severity of depression and anxiety in patients with a physical symptomatology. The scale mainly includes items that are not related to somatic symptoms of depression.

The Langer Mindfulness Scale will be used to assess the level of meditation attunement. This is a 14-item questionnaire that assesses three domains associated with mindful thinking: novelty seeking, engagement, and novelty producing [26]. An individual who seeks novelty perceives each situation as an opportunity to learn something new. An individual who scores high in engagement is likely to notice more details about his or her specific relationship with the environment.

The Medical Outcomes Study sleep measure will be used to investigate the quality of sleep [27]. This measure is composed of ten items, recording on a Likert scale the frequency of occurrence in the previous 4 weeks of symptoms and difficulties typically affecting sleep and daytime activities of patients with chronic illnesses.

Rates of refusal to join the interventions groups after randomization, as well as drop-out rates, will be assessed. The software used will keep an access log, recording the number and duration of accesses, together with a track of used tasks.

The psychological assessment phase will begin with the baseline evaluation, at month 6, with the beginning of the intervention phase. It will last 15 months (6 months after the end of the recruitment phase, to allow follow-up assessments).

#### Sleep and daily activities instrumental assessments

Actigraphy is a non-invasive method of monitoring cycles of rest and activity; a sensor unit can be worn to measure gross motor activity. The actigraphic sensor unit is similar to a watch and continually records movement.

### Analysis

The general linear model will be used to test interaction effects between treatments (experimental versus control groups) and over time (three repeated measures). Six models will be built, one of each primary outcome measure (quality of life, anxiety, and depression) and separately for patients and caregivers. Type I error inflation due to multiplicity of tests will be controlled by the correction of critical alpha (0.05/6 = 0.008). Violations of the parametric model assumptions will be tested and explored with ad-hoc techniques. If violations are detected, robust methods, data manipulation, or statistical corrections will be applied accordingly. An intention-to-treat analysis approach will be used. Missing data will be imputed with multiple imputation techniques [28]. According to power analysis calculation (G*Power), a total sample size of 110 patients (and caregivers) will allow the detection of small interaction effects (*η*^2^ = 0.01) with a 0.85 statistical power, alpha = 0.05 and an assumed nonsphericity correction (*ε*) of 0.75. Significant interaction effects will be followed by contrast analysis. A similar analysis will be conducted for the actigraphic data, with a reduced sample size, enabling the detection of a small-medium interaction effect under the same conditions.

The mediation effect of the meditation level will be examined by adding a within-subjects factor to the general linear models and the interactions with the treatment effect will be explored according to the guidelines of Kraemer *et al.* [29]. In addition, moderation effects by sex, age, and number of accesses to the software will be examined by adding the relative between-subjects factors into the general linear models and the interactions with the treatment effect will be explored according to the same guidelines [29].

Data analysis will be performed with R and SPSS software.

Data collection and analysis will begin at month 1, and will last 21 months, 1 month after the last follow-up data gathered.

## Discussion

Consistent with previous efforts that used new technology platforms to support psychological interventions [17, 18], the aim of this study is to investigate the impact of an MS-specific telemedicine meditation intervention on the quality of life of people with MS and their caregivers. Specifically, the main aim of this study is to create and test a multimedia software package that can teach meditation at home, following the specific requirements of people with MS, in order to improve quality of life and quality of sleep and reduce fatigue of patients with MS and their caregivers. We also aim to gain insight on the efficacy of a telemedicine meditation intervention on a range of important outcomes, giving a greater understanding of the underlying processes of change.

We anticipate some study limitations or threats to the protocol efficacy. The first is related to the virtual nature of the experience. The online platform could be an excessively awkward intermediary, and might reduce the feasibility of the experience compared with meditation in a group. Moreover, the synchronous communication that characterizes video-chat interactions could be slowed down by overlapping in turn-taking, or by delays and problems of internet connections.

Despite possible limits, this study has several key strengths. It consists of a meditation intervention specifically based on MS features and needs, aimed at improving the management of MS symptoms, such as numbness, fatigue, or tingling. The intervention is addressed not only to patients but also to caregivers, who may wish to obtain benefits from a meditation intervention. Moreover, the protocol is designed to reduce participation barriers, either for practical reasons (e.g., work activities) or related to physical disabilities that prevent movement. Finally, the efficacy of the experimental condition is compared with an active intervention (psycho-education group) to provide a reliable estimation of changes promoted by this specific intervention, reducing placebo and Hawthorne effects.

The findings of this study could be most relevant in improving the quality of life of people with MS and their caregivers. Scientific literature suggests that meditation improves the psychological well-being of MS patients [16]. However, participation in meditation groups can be hindered for practical reasons (e.g., work activities) or because of limitations to the physical functions. The software developed in the project will allow many people with MS (on a worldwide scale, after language translations) to access a specific meditation protocol. Moreover, caregivers of patients with MS often report distress and psychological suffering. Few studies have investigated the impact of psychological interventions on them. The inclusion of caregivers in the protocol allows us to verify whether this treatment improves their quality of life.

This intervention has the potential to offer an easy-access, freely available online resource that can provide an evidence-based approach to improve quality of life of people diagnosed with MS and their caregivers. This protocol describes the development of the intervention and the feasibility of evaluating it using a randomized controlled trial design. If the project’s hypotheses are confirmed, both patients with MS and caregivers will be able to use, at their own convenience, a specific meditation protocol, with a potentially great impact in this field.

## Trial status

Recruitment is ongoing.

## Abbreviation

MS: multiple sclerosis.
